# 

**Published:** 1816-03

**Authors:** 


### CORRESPONDENCE.

Case of Cardiac, Hepatic, and Pulmonic Disease, with Appearances on Dissection, in our next. Willan on Porigo and Impetigo, is under Review. Whateley's "Practical Observations on Ulcers of the Legs," have been received.

